# Graph Neural Network Modelling of Pre-Diagnostic Serum Proteomics for Pancreatic Cancer Detection

**DOI:** 10.3390/diagnostics16203283

**Published:** 2026-10-09

**Authors:** Alexey Zaikin, Aleksandra Gentry-Maharaj, Sophia Apostolidou, Usha Menon, Arseniy Trukhanov, Janna G. Oganezova, Harry J. Whitwell, Oleg Blyuss

**Affiliations:** 1 Research Center in Artificial Intelligence, Institute of Information Technologies, Mathematics and Mechanics, Lobachevsky State University, Nizhny Novgorod 603022, Russia; 2 Department of Mathematics and Women’s Cancer, University College London, London WC1E 6BT, UK; 3 Institute for Cognitive Neuroscience, University Higher School of Economics, Moscow 101000, Russia; 4 Department of Women’s Cancer, EGA Institute for Women’s Health, University College London, London WC1E 6AU, UK; 5 UCL Innovative Clinical Trials Unit, University College London, 90 High Holborn, London WC1V 6LJ, UK; 6 Mriya Life Institute, National Academy of Active Longevity, Moscow 124489, Russia; 7 Academician A.P. Nesterov Department of Ophthalmology of the Institute of Clinical Medicine, Pirogov Russian National Research Medical University, Moscow 117997, Russia; 8 National Phenome Centre and Imperial Clinical Phenotyping Centre, Department of Metabolism, Digestion and Reproduction, IRDB, Imperial College London, London W12 0NN, UK; 9 Section of Bioanalytical Chemistry, Division of Systems Medicine, Department of Metabolism, Digestion and Reproduction, Imperial College London, Burlington Danes Building, London W12 0NN, UK; 10 Centre for Cancer Screening, Prevention and Early Diagnosis, Wolfson Institute of Population Health, Queen Mary University of London, London EC1M 6BQ, UK; 11 Department of Paediatrics and Paediatric Infectious Diseases, Institute of Child’s Health, I.M. Sechenov First Moscow State Medical University ( Sechenov University), Moscow 119991, Russia

**Keywords:** graph neural networks, early cancer detection, proteomics, pancreatic cancer, PDAC, CA19-9, synolitic networks, biomarker discovery, UKCTOCS, machine learning

## Abstract

**Background/Objectives:** Pancreatic ductal adenocarcinoma (PDAC) has exceptionally high mortality, largely because of late diagnosis, while established blood biomarkers such as CA19-9 have limited sensitivity for early detection. We evaluated whether modelling serum proteomic measurements as supervised pairwise statistical graphs using synolitic graph neural networks (SGNNs) could provide a useful framework for pre-diagnostic PDAC classification. **Methods:** We analysed 217 pre-diagnostic serum samples from UKCTOCS, comprising 100 PDAC samples from 75 women and 117 control samples, profiled for 97 proteins. Samples were divided into a development set collected 0–1 year before diagnosis (*n* = 110) and a temporal holdout set collected 1–2 years before diagnosis (*n* = 107); 25 PDAC participants contributed samples to both windows. The final SGNN used fold-internal mutual-information selection of 30 proteins, pairwise RBF-SVM-derived edge information, minimum-connected sparsification, and a 10-member GATv2 ensemble per cross-validation fold. Conventional machine-learning comparators were tuned using development data only. **Results:** The SGNN achieved a mean 5-fold development ROC-AUC of 75.33 ± 10.43%, lower than the tuned Random Forest (81.67%); the other tuned comparators achieved 77.67% (XGBoost), 77.17% (logistic regression), and 73.17% (SVM). Averaging predictions across the five-fold-specific SGNN ensembles yielded a temporal-holdout ROC-AUC of 64.35% (95% CI 52.6–74.6%). In the participant-independent subset of 25 PDAC cases not represented in development and 57 controls, ROC-AUC was 66.04% (95% CI 52.56–78.88%). **Conclusions:** SGNNs provide a feasible graph-based representation of pre-diagnostic proteomic data but did not outperform optimised conventional machine-learning methods within development cross-validation. The temporal and participant-independent performance estimates warrant further investigation in larger fully independent cohorts. A graph-specific predictive advantage was not directly tested—no such advantage was demonstrated in this dataset—and the clinical utility has not been established.

## 1. Introduction

Pancreatic ductal adenocarcinoma (PDAC) has a particularly poor prognosis and remains a major cause of cancer mortality, ranking seventh globally and fourth among cancer-related deaths in Western countries; five-year survival remains below 10% [1,2,3]. Its burden is expected to rise further, with projections indicating that PDAC mortality may surpass that of several other common cancers by 2030 [2,4]. Early detection is difficult because initial disease is often clinically silent and the anatomical location of the pancreas limits opportunities for early recognition [5,6]. Consequently, many patients present with stage III or IV disease, when curative surgical resection is no longer feasible, and fewer than one in five are diagnosed while resection remains possible [7,8].

Outcomes are considerably more favourable when pancreatic neoplasia is identified before progression to advanced disease, including at the stage of pre-invasive lesions or small localised tumours (<2 cm) [9]. However, the low incidence of PDAC and a general-population lifetime risk of approximately 1.3% make screening of asymptomatic average-risk adults impractical. Surveillance therefore focuses on groups with substantially elevated risk, including individuals with genetic susceptibility, a strong family history, or high-risk pancreatic cysts, for whom a lifetime risk of at least 5% is considered sufficient to justify surveillance [5,10,11]. These considerations make earlier case identification a major goal of efforts to improve PDAC outcomes [12,13].

Among circulating biomarkers used in PDAC care, carbohydrate antigen 19-9 (CA19-9) is the most established, but its value for early detection is limited. Reported diagnostic performance is approximately 80% for both sensitivity and specificity, with an AUC of around 0.82, and performance is weaker in stage I/II disease [14,15,16]. In stage I tumours, sensitivity may be as low as 50%. CA19-9 can also be elevated in benign conditions, such as chronic pancreatitis, biliary obstruction, and other gastrointestinal disorders, reducing its specificity for pancreatic cancer [16,17]. In addition, approximately 5–10% of the population lacks the Lewis antigen required for CA19-9 expression and therefore cannot generate an informative CA19-9 signal [18]. These limitations have motivated evaluation of CA19-9 in combination with additional biomarkers rather than as a standalone screening or diagnostic test [15,19].

Efforts to develop blood-based markers for PDAC now span protein signatures, microRNAs, circulating tumour cells, and DNA methylation assays, although most candidates still lack validation in cohorts representative of their intended clinical use [20,21]. Blood-based multi-cancer assays, including CancerSEEK and Galleri, have also been developed to detect signals from several tumour types [22,23]. These platforms use combinations of circulating molecular features, including somatic mutations, protein measurements, and cancer-associated DNA methylation patterns. CancerSEEK reported 72% sensitivity for pancreatic cancer (stages I–III) at approximately 99% specificity, while a methylation-based multi-cancer early detection test reported 83.7% sensitivity for PDAC at 99.5% specificity [22,23,24]; however, sensitivity varies across cancer types, and additional larger validation studies are needed. In a complementary approach, our group has previously pursued two related lines of work using the same Olink Oncology II panel. Nené et al. [25] analysed prospectively collected pre-diagnostic UKCTOCS serum samples and developed a stacked ensemble classifier that achieved AUC 0.91 for samples collected up to one year before diagnosis and 0.85 for samples up to two years before diagnosis. Subsequently, Ney et al. [26] developed a diagnosis-specific ensemble learning biomarker signature comprising 44 serum protein and clinical covariates, trained on symptomatic cohorts from the ADEPTS and UKCTOCS studies, that achieved AUC 0.95 in a held-out validation set for distinguishing diagnosed PDAC cases from controls. These studies demonstrate the value of ensemble methods and multi-marker panels for PDAC detection but address different clinical scenarios (pre-diagnostic screening vs. diagnostic confirmation).

High-throughput serum proteomic platforms can quantify hundreds to thousands of proteins simultaneously, creating substantial opportunities for multivariable biomarker discovery. Measuring many proteins concurrently provides a broad multivariable representation of circulating biological state that cannot be obtained from a single-marker assay. However, this technological capability introduces analytical challenges in clinical settings where sample sizes are typically modest. The dataset used in the present study comprises *p* = 97 protein features measured across *n* = 217 patient samples (*n* = 110 in the development set). While *p* does not vastly exceed n at the dataset level, within individual cross-validation training folds (approximately 88 samples), the feature-to-sample ratio approaches unity. This small-sample, moderately high-dimensional setting increases the risk of overfitting, particularly when complex, nonlinear relationships are modelled, and demands careful cross-validation with strict prevention of information leakage [27].

A systems-level view of cancer emphasises coordinated disturbances across interacting molecular processes rather than isolated abnormalities in individual proteins [28]. Under this view, an early disease signal may arise from distributed and nonlinear changes across several proteins rather than from a large change in any single marker. Several proteins identified in prior PDAC biomarker studies—including VWF, THBS2, CEACAM5, and EGF—participate in interconnected cancer-associated signalling cascades, such as the PI3K–AKT, ERBB, and MAPK pathways [26,29]. These considerations motivate evaluation of analytical frameworks that represent pairwise statistical information between protein measurements explicitly, while recognising that such model-derived relationships are not themselves evidence of biological protein–protein interaction. Tissue-based molecular biomarkers, including PD-L1 expression, have also been investigated in pancreatic cancer in relation to tumour phenotype and prognosis [30].

Relational data can be modelled using graph neural networks (GNNs), which learn representations from features associated with graph nodes and their connections [31]. GNNs update node representations by repeatedly combining information from neighbouring nodes, thereby allowing each node representation to incorporate both its own attributes and information from the surrounding graph structure. GNNs have demonstrated remarkable success in computational biology applications, including drug discovery [32], protein structure prediction, and cell-type classification from single-cell data [33]. Applying a GNN to proteomic data provides a modelling strategy in which supervised pairwise statistical information is represented explicitly alongside individual protein measurements. In the present study, these graph edges are model-derived classification quantities rather than direct measures of biological protein–protein interaction; the analysis therefore evaluates the predictive utility of this representation rather than attempting to infer biological network structure.

In this study, we evaluate a supervised graph representation of pre-diagnostic serum proteomic measurements for PDAC classification and benchmark it against conventional machine-learning approaches using development-set cross-validation and a temporally separated holdout cohort. We employed the analytical framework based on synolitic networks and GNNs [34] to model high-dimensional proteomic data from the UK Collaborative Trial of Ovarian Cancer Screening (UKCTOCS) [35], using samples from PDAC cases and matched healthy controls. The UKCTOCS-derived proteomic measurements analysed here are from the same participant cohort as our prior ensemble learning study [25]. However, the two studies differ in temporal stratification, study endpoints, and analytical approach: the prior work [25] pooled samples from both pre-diagnostic temporal windows without the same temporal stratification used here, whereas the present study applies a single, consistent temporal stratification using proteomic data alone, precluding direct numerical comparison of performance. To our knowledge, this is the first application of synolitic graph neural networks to pre-diagnostic PDAC proteomic data.

## 2. Materials and Methods

### 2.1. Ethics Statement

The UKCTOCS protocol and use of samples in subsequent ethically approved investigations were approved by the Joint UCL/UCLH Research Ethics Committee A (ref. 05/Q0505/57). All participants had provided consent permitting use of their samples for the trial and approved secondary research. The analytical dataset supplied to the study team contained no directly identifying patient information.

### 2.2. Study Cohort and Samples

Serum specimens for this nested case–control analysis were obtained from the UK Collaborative Trial of Ovarian Cancer Screening (UKCTOCS) [35,36], a randomised controlled trial that recruited 202,638 post-menopausal women aged 50–74 between 2001 and 2005. UKCTOCS was supported by the Medical Research Council (G9901012 and G0801228), CRUK and the Department of Health, with additional support from The Eve Appeal. Participants allocated to the multimodal screening arm underwent annual blood testing, with residual serum collected and stored according to a standardised operating procedure [37]. Cancer incidence and mortality were ascertained through linkage to national records using participants’ NHS identifiers. Women who subsequently developed PDAC were identified from cancer-registry and mortality records using ICD-10 codes C25.0, C25.1, C25.2, C25.3 and C25.9. Diagnostic confirmation was additionally sought through questionnaires sent to general practitioners and hospital consultants and through Hospital Episode Statistics.

The analytical dataset comprised 217 serum samples, including 100 pre-diagnostic PDAC samples and 117 matched control samples. Samples with a confirmed diagnosis of pancreatic cancer were temporally stratified into 50 collected within 0–1 year prior to diagnosis and 50 collected 1–2 years prior to diagnosis. Control samples were obtained from women without a recorded cancer diagnosis and were matched to case samples with respect to age, collection timing, and recruitment centre, thereby limiting potential differences associated with sample handling or storage. From this set, 117 controls were selected (60 controls matched to the 0–1 year cohort and 57 controls matched to the 1–2 year cohort). This temporal stratification enabled cross-validated model development on near-diagnostic samples (the development set, *n* = 110) and assessment of early detection capability on the more challenging 1–2 year pre-diagnostic window (the holdout set, *n* = 107).

Because the UKCTOCS cohort collected longitudinal annual samples from PDAC cases, individual women may contribute samples to both temporal windows. An analysis of sample identifiers revealed that 25 of the 50 cancer cases in the development set (0–1 year) are from the same women who also contributed a sample to the 1–2 year holdout set; the remaining 25 cancer cases in each set are from distinct individuals. All 117 controls are unique across the two sets. Within the development set, each participant contributes exactly one sample, so the 5-fold cross-validation is participant-independent. However, the temporal holdout is only partially participant-independent because the earlier 1–2-year samples from 25 PDAC participants are evaluated after their later 0–1-year samples contributed to model development. To examine whether shared participant-specific proteomic characteristics influence holdout performance, we additionally performed a sensitivity analysis restricted to the 25 holdout PDAC cases from women not represented in the development cohort, together with all 57 holdout controls. Results of this participant-independent analysis are reported in Section 3.4.

The proteomic dataset analysed in the present study is derived from the same UKCTOCS-based sample set previously employed in our ensemble learning work [25,26]. The present study applies a fundamentally different analytical framework—synolitic graph neural networks—to this dataset. However, direct numerical comparison with the prior studies is not appropriate because of differences in temporal stratification (the prior pre-diagnostic study [25] pooled both time windows; the diagnosis-specific study [26] included symptomatic samples and clinical covariates), study endpoints, and analytical approach.

### 2.3. Proteomic Data Generation and Preprocessing

All serum specimens were allocated randomly for laboratory analysis. Five candidate markers were quantified using single-analyte assays. CA19-9 was measured with the Mucin PC/CA19-9 ELISA Kit (Alpha Diagnostic International Inc., San Antonio, TX, USA) after 1:4 serum dilution. CA125/MUC16 was quantified on a Cobas E411 analyser using the Cobas CA125 II CLIA system, CA125 II calibrators, and PreciControl Tumour Marker material (Roche Diagnostics, Mannheim, Germany/Fujirebio Diagnostics Malvern, PA, USA). VWF was analysed with the Abcam Human Von Willebrand Factor ELISA (Abcam, Cambrdge, UK) at a 1:100 dilution. IL6ST/IL6RB was measured using the Quantikine human soluble gp130 assay (R&D Systems, Minneapolis, MN, USA), and THBS2/TSP2 using the Quantikine Human Thrombospondin-2 assay (R&D Systems, Minneapolis, MN, USA) at a 1:10 dilution. Measurements were obtained from single assay runs for each sample; observations falling below the quantifiable assay range were recorded as “low”.

The candidate-marker measurements were combined with data for 92 additional cancer-related proteins quantified using the Olink Oncology II multiplex panel (Olink Proteomics, Uppsala, Sweden). The panel includes proteins spanning several functional classes relevant to cancer, including tumour-associated antigens, signalling and growth factors, receptors, angiogenesis-related proteins, and adhesion molecules. Quantification is based on Proximity Extension Assay technology [38], and Olink measurements were supplied as log2-transformed normalised protein expression (NPX) values. Combining the five single-analyte measurements with the 92 Olink measurements produced 97 protein variables for each serum sample. After quality control, the proteomic dataset contained no missing values.

Importantly, the dataset combines Olink NPX measurements on a log2 scale with ELISA/ECLIA markers in assay-specific units. To address these heterogeneous scales, all features were standardised (zero mean, unit variance) using a StandardScaler fitted exclusively on the training data within each cross-validation fold. The same learned transformation was then applied to the corresponding validation fold and holdout samples, ensuring no information from test or holdout data influenced the scaling parameters. This fold-internal standardisation is critical for scale-sensitive methods, including SVM, logistic regression, and the pairwise classifiers used in graph construction.

### 2.4. The Synolitic Graph Neural Network (SGNN) Framework

**Figure 1:**
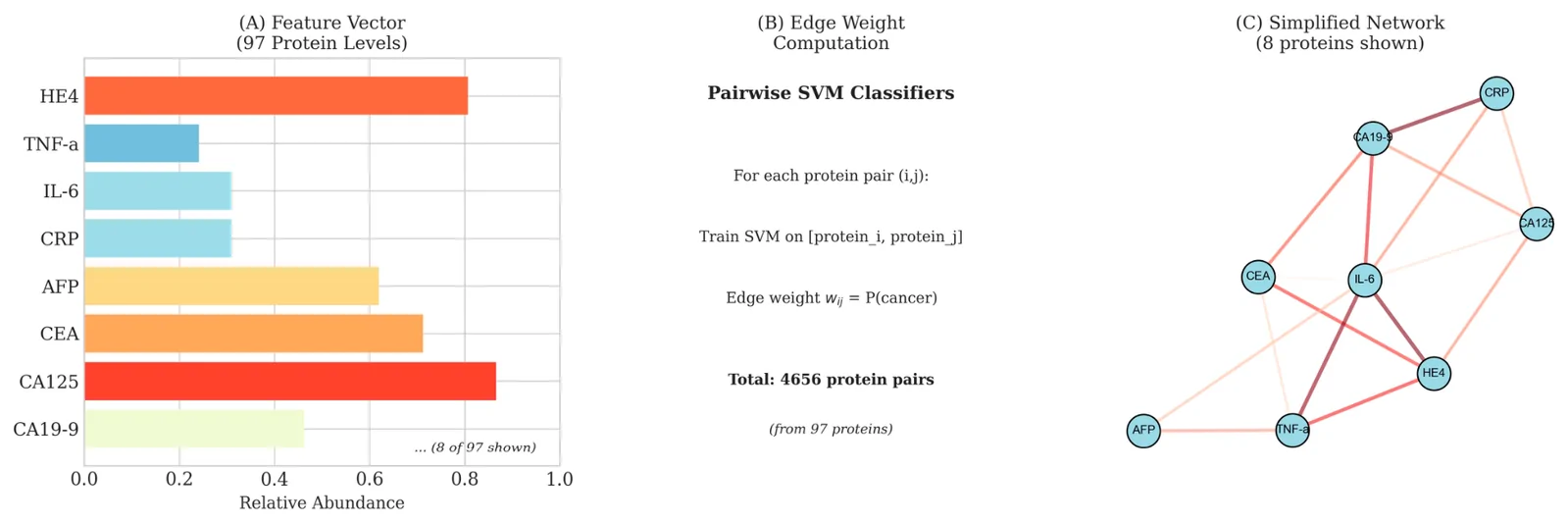
Schematic overview of the synolitic graph-construction methodology. (**A**) Serum protein measurements form the input feature vector (eight proteins shown for clarity). (**B**) Pairwise RBF-SVM classifiers trained exclusively on the relevant training data generate sample-specific cancer-class probabilities for each protein pair. These supervised probabilities contribute to the graph edge representation and do not represent biological protein–protein interaction. (**C**) Simplified sample-specific graph representation showing eight illustrative protein nodes.

To address the analytical challenges of the proteomic dataset (*p* = 97, *n* = 110 in the development set), we employed a synolitic graph neural network (SGNN) framework [34]. In this study, ‘synolitic’ refers to the integration of multiple supervised pairwise statistical relationships into a sample-specific graph representation. Proteins constitute graph nodes, while model-derived quantities from pairwise classifiers provide edge information. The resulting graphs are computational representations designed for classification and should not be interpreted as physical or biological protein–protein interaction networks. These elements help reduce overfitting risk in the small-sample, moderately high-dimensional setting typical of clinical proteomic studies [27,39]. Accordingly, the conventional vector of protein measurements is recast as a sample-specific graph for subsequent GNN analysis (Figure 1).

**Figure 2:**
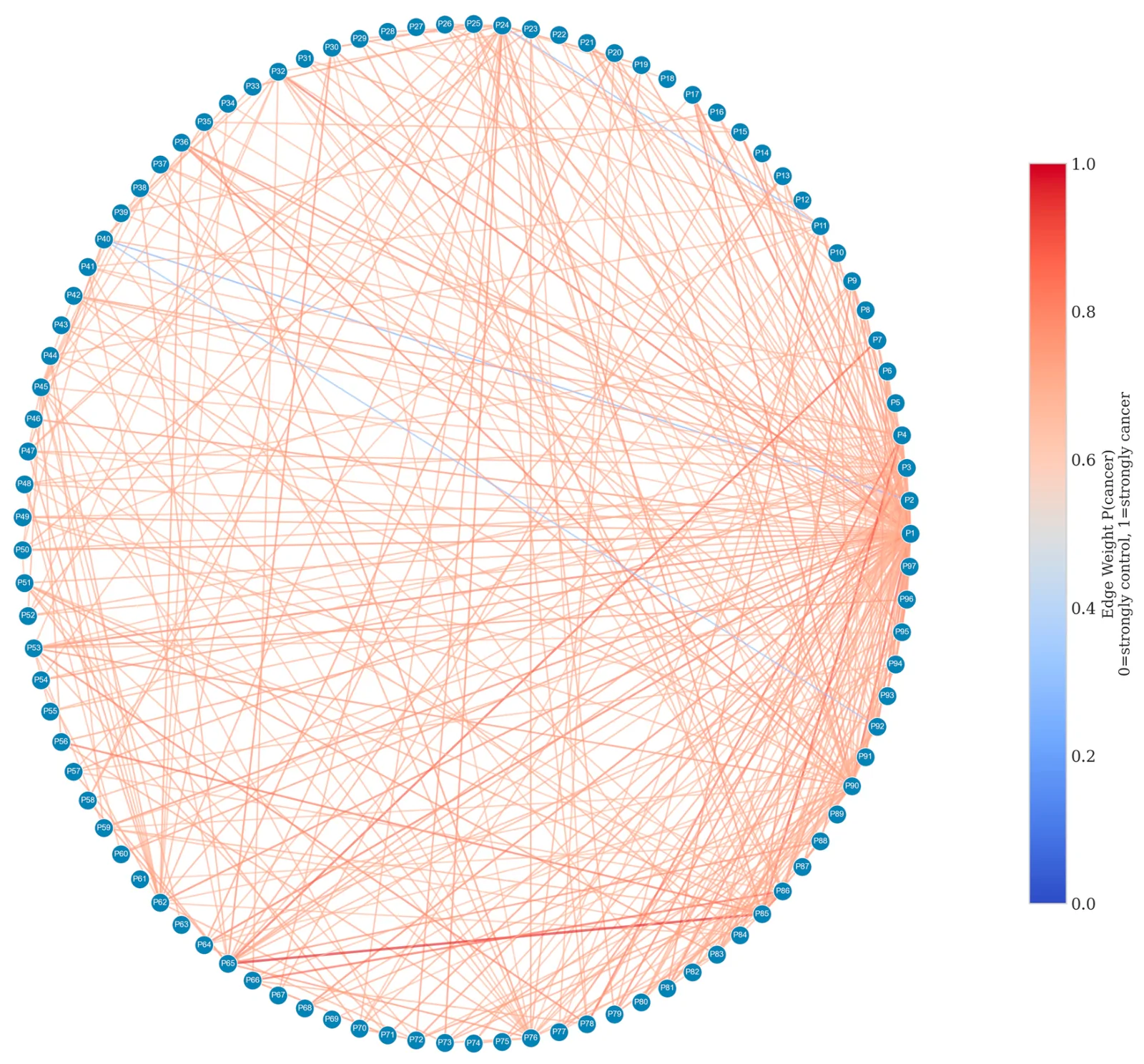
Illustrative minimum-connected graph from the standard 97-protein SGNN pipeline, shown to visualise the underlying pairwise-SVM graph-construction procedure. In the standard pipeline, sparsification selects the largest threshold on ∣*p_ij_* − 0.5∣, where *p_ij_* is the pairwise RBF-SVM cancer-class probability, for which the graph remains connected. Edge colours show the corresponding model-derived cancer-class probabilities. These edge quantities are supervised classifier outputs and should not be interpreted as biological protein–protein interactions. The final improved SGNN applies an analogous connectivity constraint to the hybrid edge-ranking quantity ∣*w_ij_* − 0.5∣ after fold-internal feature selection.

Graph Construction: Before sparsification, each sample was represented by a fully connected candidate graph over the proteins retained by the corresponding pipeline. The standard SGNN used all 97 proteins, giving 4656 unordered candidate edges. In the improved pipeline, mutual-information feature selection was performed within each outer training fold; the final reported configuration retained 30 proteins, giving 435 unordered candidate edges. An illustrative minimum-connected graph generated using the standard 97-protein SGNN pipeline is shown in Figure 2.

Edge Weight Definition: For each protein pair *i*,*j*, an RBF-kernel support vector machine (*SVC*, *C* = 1.0, *γ* = *scale*, *probability* = *True*) was trained using only the two standardised protein measurements in the relevant training data. For each sample, the classifier generated a cancer-class probability *p_ij_*. This is a supervised sample-level classification score derived from two protein measurements and is not a measure of protein dependence, biological interaction, or physical connectivity. In the improved pipeline, the pairwise classifier was fitted using training data only and subsequently applied to validation, outer-test, and temporal-holdout samples without refitting.

### 2.5. Graph Feature Engineering and GNN Architecture

Node Feature Augmentation: In the improved SGNN pipeline, each protein node was represented by six features: (1) the standardised protein value *z_i_*; (2) the normalised node index *i*/*m*, where *m* is the number of selected proteins; (3) normalised degree; (4) normalised weighted strength; (5) closeness centrality; and (6) betweenness centrality. All four graph-topological quantities were computed after sparsification. Degree, closeness, and betweenness were calculated from the unweighted sparsified topology, while node strength was calculated using the retained hybrid edge weights. No 1/*w_ij_* distance transformation was applied in the improved pipeline. These quantities are treated as algorithmic descriptors of the model-derived graph and are not interpreted as measures of biological protein interaction.

GNN Models: We evaluated several GNN architectures, including the Graph Convolutional Network (GCN) [40] and the Graph Attention Network v2 (GATv2) [41]. The two architectures differ principally in how information from adjacent nodes is combined. In the GCN, neighbouring node features are propagated according to the graph-convolution operation, whereas GATv2 estimates attention coefficients during training so that contributions from different neighbours can vary. This adaptive weighting may be useful when the predictive relevance of graph neighbours differs between proteins and samples.

Training Details: The final improved SGNN used a GATv2 architecture with three message-passing layers, 64 hidden channels, four attention heads, three-dimensional edge attributes, residual connections, batch normalisation, and dropout of 0.3. The edge attribute vector was [*p_ij_*, *w_ij_*, |*p_ij_* − 0.5|]. Optimisation used AdamW with a 0.005 learning rate and 10^−3^ weight decay, cosine-annealing learning-rate scheduling, gradient clipping at norm 1.0, batch size of 16, and inverse-frequency class-weighted cross-entropy. Gaussian noise with SD 0.05 was added to node features as augmentation on alternate epochs. Models were trained for up to 300 epochs, with a minimum of 30 epochs and early-stopping patience of 40.

Within each outer training fold of 88 samples, the internal validation subset used for checkpoint selection was generated using StratifiedShuffleSplit (n_splits = 1, test_size = 0.15, random_state = 42) applied exclusively to that training fold. This produced 74 model-training samples and 14 internal-validation samples (6 PDAC cases and 8 controls) in every outer fold. This split was performed before any supervised fitting. Within each outer fold, the StandardScaler, mutual-information feature selection, and all 435 pairwise SVM classifiers were fitted on the 74 model-training samples only; the 14 internal-validation samples were transformed using these fitted components and were used solely for early stopping and checkpoint selection. The internal-validation samples therefore did not contribute to feature selection or graph-edge construction in the reported model. The checkpoint with the highest internal-validation ROC-AUC was retained. Neither the outer CV test fold nor the temporal holdout was used for training, early stopping, or checkpoint selection.

For GATv2 attention pooling, attention weights were normalised separately over the nodes belonging to each graph using the PyTorch v.2.10.0 Geometric batch-assignment vector before weighted-sum pooling. This ensures that the prediction for an individual graph is invariant to the composition of the mini-batch. Regression testing confirmed agreement of predictions across batch sizes 1, 4, and 8 to within 6 × 10^−8^.

**Figure 3:**
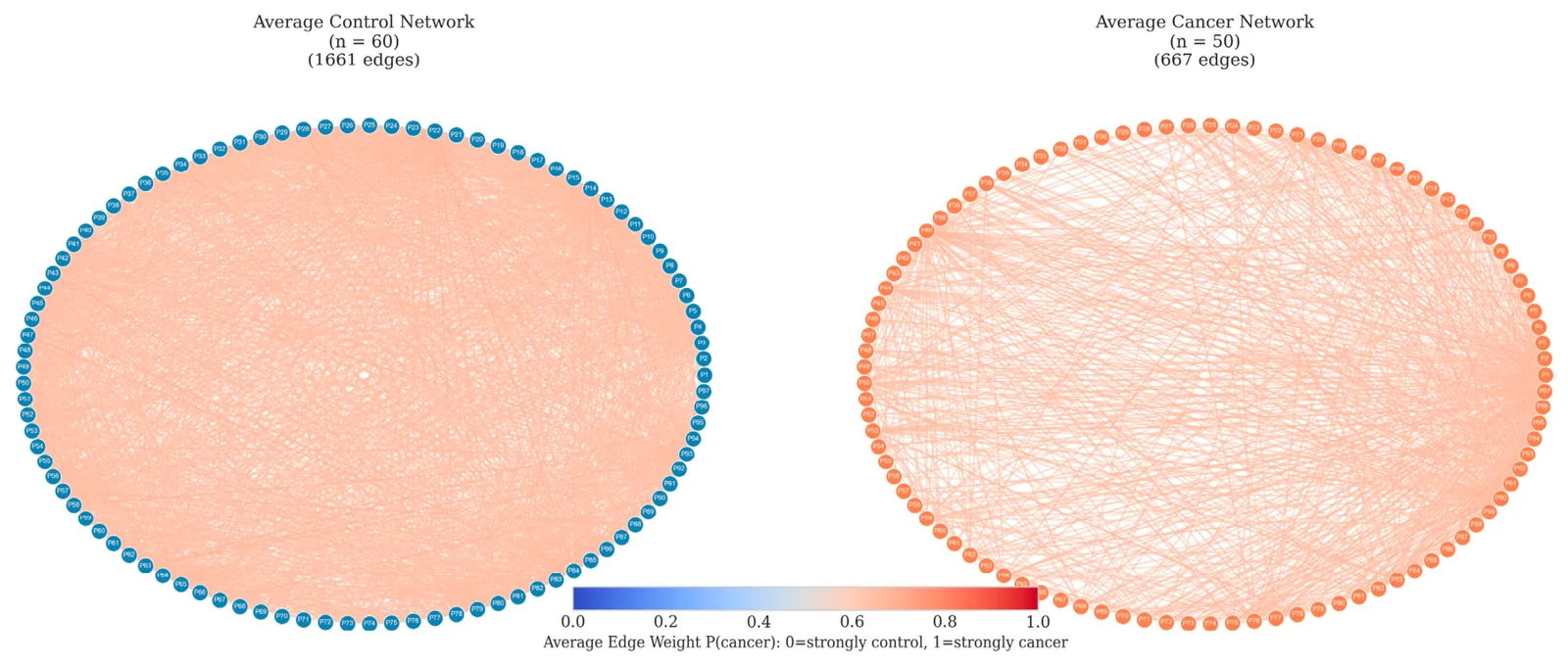
Average synolitic networks computed across all development-set samples per class. Left panel: average control network (*n* = 60, 1661 edges); right panel: average cancer network (*n* = 50, 667 edges). For each protein pair, pairwise SVM cancer-class probabilities were computed per sample and then averaged within each class. Minimally connected sparsification was applied to the averaged edge weights. The difference in edge density provides a descriptive illustration of how pairwise classifier-derived scores differ between the class-averaged graphs. It should not be interpreted as evidence of biological network rewiring or as demonstrating that this particular topological difference is responsible for GNN classification performance. Edge colours indicate average model-derived *P*(*cancer*). Because the minimum-connected threshold was determined separately for each class-averaged graph, edge counts are not directly comparable between panels, and the difference was not subjected to statistical testing; the figure is descriptive only.

Figure 2 and Figure 3 illustrate graph construction using the original 97-protein standard SGNN pipeline and are included to visualise the pairwise-SVM graph representation and sparsification procedure; they do not represent the final feature-selected model used for the principal predictive analyses.

### 2.6. Graph Sparsification

Minimum-connected sparsification was used for the final improved SGNN. For each sample, candidate edges were ranked using the following:

*q_ij_* = |*w_ij_* − 0.5| (1)

Here, *w_ij_* is the hybrid edge weight defined in Section 2.7. The largest threshold for which the retained graph remained connected was selected by binary search over the candidate thresholds. Because *w_ij_* combines a supervised class probability with a feature-similarity term, *q_ij_* is used only as an algorithmic edge-ranking criterion and is not interpreted as classifier confidence or as a measure of biological interaction.

The binary-search implementation was verified against exhaustive brute-force threshold selection on synthetic and real PDAC graphs. In the final 30-protein configuration, minimum-connected sparsification retained approximately 73–85 of the 435 candidate undirected edges per graph (17–20%).

For the standard SGNN pipeline, where the edge score is the raw pairwise SVM probability *p_ij_*, the corresponding ranking criterion is |*p_ij_* − 0.5|.

### 2.7. Improved SGNN Pipeline with Feature Selection

The improved pipeline incorporated fold-internal feature selection, hybrid edge information, and ensemble GNN training. Mutual-information feature selection (mutual_info_classif, random_state = 42) was fitted exclusively within each outer training fold. Candidate feature-set sizes of 20, 30, and 40 proteins were evaluated as pragmatic dimensionality reductions in the original 97-protein panel; these values were not intended to represent biologically established optimal panel sizes.

For each selected protein pair, the pairwise RBF-SVM cancer-class probability *p_ij_* was combined with within-sample feature similarity to define the following:

(2) $$w_{\mathrm{ij}}=0.7{\;p}_{\mathrm{ij}}+0.3{\left(1+\left|z_{i}-z_{j}\right|\right)}^{-1}$$

Here, *z_i_* and *z_j_* are the standardised protein values for the sample. The coefficients 0.7 and 0.3 were fixed a priori and were not optimised. GATv2 received the three-dimensional edge attribute vector [ *p_ij_*, *w_ij_*, | *p_ij_* − 0.5|].

The configuration search comprised two stages. Stage A used a full factorial design across feature count (20, 30, or 40), architecture (GATv2 or GCN), and checkpoint criterion (val_auc, val_loss, or val_auc_loss), giving 18 trained configurations. Stage B evaluated 13 one-factor-at-a-time variants around the leading Stage A configuration: learning rate 0.002 or 0.010 instead of 0.005; hidden width 32 or 128 instead of 64; dropout 0.5 instead of 0.3; two instead of three message-passing layers; weight decay 10^−2^ instead of 10^−3^; no data augmentation; internal-validation fraction 0.10 or 0.25 instead of 0.15; and ensemble sizes of five or ten rather than three, together with a repeat of the Stage A reference configuration. In total, 31 configurations were trained. Each was evaluated both as a single-model read-out and as an ensemble read-out, giving 62 reported results.

Model-configuration selection was based exclusively on mean development-set 5-fold ROC-AUC and did not use the temporal holdout. Selection was restricted to ensemble configurations. The selected model family used 30 proteins, GATv2 architecture, and validation-AUC checkpointing. Final reporting used a 10-member seed ensemble within each outer fold to reduce stochastic training variability; member probabilities were averaged within each fold. Repeated evaluations showed lower run-to-run variability for the 10-member ensemble than for single-member evaluation. Because configuration selection and performance estimation used the same development folds, the resulting CV performance remains exploratory and may be affected by selection-induced optimism. Nested cross-validation was not undertaken because the development cohort comprised only 110 samples, which would have resulted in very small inner training and validation subsets.

### 2.8. Statistical Analysis and Benchmarking

The dataset was partitioned for model training and validation using a temporal stratification strategy. The development set consisted of the 50 samples collected less than one year before clinical diagnosis together with 60 matched control samples (*n* = 110). This set was used for model training, cross-validated evaluation, and all model selection decisions. The early-detection holdout set comprised 50 samples collected one to two years prior to PDAC diagnosis together with 57 matched control samples (*n* = 107), providing a temporal holdout assessment of the model’s early-detection capability. Because 25 of the 50 holdout PDAC cases were longitudinal samples from women represented in development, this complete holdout is temporally separated but only partially participant-independent. A sensitivity analysis therefore repeated evaluation after restricting the PDAC cases to the 25 women not represented in development.

To provide a more comparable benchmark, four conventional machine-learning approaches were tuned using development data only and the same outer cross-validation folds. Feature count (20, 30, 40, or all 97 proteins) was included as a tuning dimension; where fewer than 97 proteins were used, mutual_info_classif feature selection was fitted within the relevant training fold. The search comprised 180 configurations: XGBoost (64 configurations after crossing with feature count)—max_depth {3,6}, learning_rate {0.05,0.1}, n_estimators {100,300}, subsample {0.8,1.0} [42]; Random Forest (48 configurations)—n_estimators {100,500}, max_depth {5,10,None}, max_features {sqrt,0.3} [43]; RBF-SVM (36 configurations)—*C* ∈ {0.1, 1, 10}, gamma {scale,0.01,0.1}; logistic regression (32 configurations)—*C* ∈ {0.01, 0.1, 1, 10}, with L2/LBFGS or L1/liblinear. By comparison, the SGNN search comprised 31 trained configurations (Section 2.7). For each model class, the configuration with the highest mean development CV ROC-AUC was selected. The temporal holdout was not used for hyperparameter or feature-count selection.

For the principal SGNN temporal-holdout analysis, each of the five outer-fold models comprised a 10-member seed ensemble. Member probabilities were first averaged within each fold. The resulting five-fold-level probabilities were then averaged for each holdout participant, giving one final prediction per participant. ROC-AUC was calculated once from this 107-participant prediction vector. The same prediction vector was used for the participant-independent and overlapping-participant sensitivity analyses.

Uncertainty was quantified using 2000 participant-level stratified bootstrap replicates, resampling cases and controls separately, and reporting 95% percentile confidence intervals. The holdout labels were not used for standardisation, feature selection, pairwise-SVM fitting, GNN training, early stopping, hyperparameter selection, or model-configuration selection.

Where mean ± SD across the five-fold-trained models is additionally reported for descriptive purposes, this SD represents variation among models trained on different development subsets and is not a confidence interval for population-level holdout performance.

ROC-AUC was the primary discrimination metric throughout the analysis. Development-set results are reported across the five outer folds, while temporal-holdout uncertainty for the principal SGNN model is reported using participant-level bootstrap confidence intervals.

As an exploratory clinical reference, raw CA19-9 (no model training; values below the standard curve set to half the lowest measured value) was evaluated on the same development folds and holdout sets, using the same bootstrap procedure. Paired differences in holdout ROC-AUC were estimated by resampling participants once per replicate, and sensitivity was compared at the 90th and 95th percentiles of the holdout control scores.

Analyses were performed using Python 3.13.12, PyTorch 2.10.0, PyTorch Geometric 2.7.0, scikit-learn 1.8.0, XGBoost 3.2.0, NetworkX 3.6.1, NumPy 2.5.3, SciPy 1.17.1 and pandas 2.3.3, on macOS 15.7.3 using an Apple M4 Max workstation (16 CPU cores, 128 GB unified memory) and the PyTorch Metal Performance Shaders backend. A complete five-fold run of the final 30-protein, 10-member GATv2 ensemble required approximately 8–10 min; approximately 95% of this time was GNN training. The complete two-stage SGNN configuration search required approximately 1 h 38 min, while the 180-configuration conventional-model search required approximately 2.2 min.

## 3. Results

### 3.1. Cohort Characteristics

The study cohort consisted of 217 serum samples, including 100 samples from individuals who were subsequently diagnosed with PDAC (50 in the 0–1 year window, 50 in the 1–2 year holdout window) and 117 samples from matched healthy controls. Because the UKCTOCS cohort comprises longitudinal samples, 25 of the 50 PDAC cases contributed one sample to each temporal window, yielding 75 unique cancer patients across the two sets. All 117 controls were unique individuals. All participants were post-menopausal women recruited to UKCTOCS [35,36]. The proteomic data of each sample comprised 97 features: 92 proteins from the Olink Oncology II panel (reported as normalised protein expression, NPX, on a log2 scale) and 5 additional ELISA/ECLIA-measured markers (CA19-9, CA125, VWF, IL6ST, THBS2) in assay-specific units. No missing values were present after quality control. The dataset was partitioned into a development set (0–1 year samples and matched controls, *n* = 110) used for cross-validated model training and selection, and a temporally separated holdout set (1–2 year samples and matched controls, *n* = 107). The full holdout was only partially participant-independent because 25 PDAC participants contributed samples to both temporal windows. Note that information regarding stage at eventual diagnosis was not available for this cohort, so the analysis does not distinguish early-stage from late-stage disease among the pre-diagnostic cases.

### 3.2. Model Performance Within 1 Year Before Diagnosis

Under 5-fold stratified cross-validation of the development cohort, the final reported SGNN configuration—a 30-protein GATv2 model using minimum-connected sparsification and a 10-member seed ensemble per fold—achieved a mean ROC-AUC of 75.33 ± 10.43%. This value is exploratory because model configuration was selected using the same development folds used for performance estimation. The development cross-validation and temporal-holdout performance of the final SGNN are summarised in Table 1.

**Figure 4:**
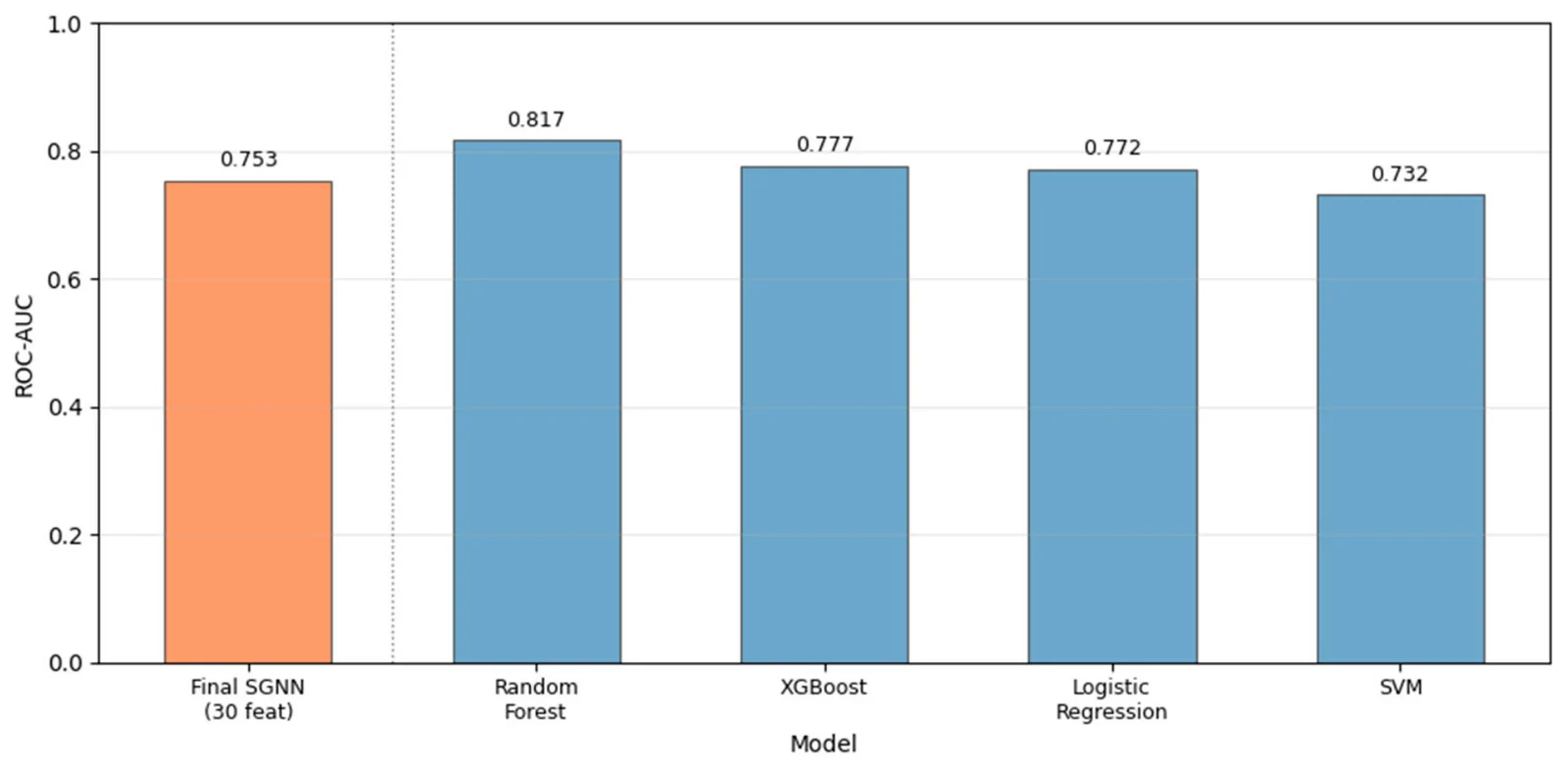
Development-set 5-fold cross-validated ROC-AUC for the final SGNN and tuned conventional machine-learning comparators. Random Forest achieved the highest mean development CV ROC-AUC (0.8167), followed by XGBoost (0.7767), logistic regression (0.7717), the final SGNN (0.7533), and SVM (0.7317). Model- and feature-selection choices were based on development data only; comparisons are exploratory.

After development-only tuning of the conventional comparators, Random Forest achieved the highest mean development CV ROC-AUC (81.67%), followed by XGBoost (77.67%), logistic regression (77.17%), the final SGNN (75.33%) and SVM (73.17%). Thus, the analysis does not demonstrate superior within-development discrimination for SGNN relative to optimised conventional machine-learning approaches. Differences between SGNN configurations were also subject to appreciable stochastic variability in this small development cohort and are therefore interpreted as exploratory rather than as evidence that a particular neighbouring configuration is intrinsically superior. The selected conventional-model configurations are detailed in Table 2, and the development-set performance comparison between the final SGNN and the tuned conventional models is shown in Figure 4.

### 3.3. Early Detection Performance (1–2 Years Before Diagnosis)

For the final SGNN, the probabilities generated by the 10-member ensemble within each of the five outer folds were first averaged within fold and then averaged across folds for each temporal-holdout participant. This produced one prediction for each of the 107 holdout samples. The resulting ROC-AUC was 0.6435 (95% participant-level bootstrap CI 0.526–0.746).

The temporal-holdout estimate should be interpreted in the context of both temporal distribution shift and participant overlap. The 1–2-year cohort differs biologically and temporally from the 0–1-year development cohort, and 25 of the 50 holdout PDAC cases are longitudinal samples from women represented in development. The effect of this overlap is examined directly in Section 3.4.

### 3.4. Participant-Independent Sensitivity Analysis

To assess the influence of participants represented in both temporal windows, we repeated the final SGNN evaluation after restricting the holdout PDAC cases to the 25 women who contributed no sample to the development cohort. Together with all 57 holdout controls, this participant-independent subset comprised 82 samples. ROC-AUC was 0.6604 (95% CI 0.5256–0.7888).

For comparison, the complementary subset comprising the 25 holdout PDAC samples from women represented in development and the same 57 controls yielded ROC-AUC 0.6267 (95% CI 0.4779–0.7558). Thus, exclusion of overlapping participants did not reduce the SGNN point estimate. However, both subgroup confidence intervals are wide and overlap substantially, so these results should not be interpreted as demonstrating a difference between overlapping and non-overlapping participants. The results of the participant-overlap sensitivity analyses are summarised in Table 3.

In an exploratory comparison, CA19-9 alone, which ranked first in mutual-information selection in all five folds and was therefore included in every SGNN graph, achieved a development CV ROC-AUC of 0.7625 ± 0.1056 and a full-holdout ROC-AUC of 0.6223 (95% CI 0.5054–0.7293). The paired difference (SGNN minus CA19-9) was +0.0212 (95% CI −0.0746 to 0.1135) in the full holdout and −0.0172 (−0.1432 to 0.0954) in the participant-independent subset. Sensitivities at the 91.2% and 96.5% specificity actually achieved were also similar (CA19-9 0.32 and 0.20; SGNN 0.30 and 0.24). The SGNN therefore performed comparably to CA19-9 alone, with no evidence of incremental discrimination. CA19-9 also showed higher discrimination in the participant-independent than in the overlapping subset (0.6775 vs. 0.5670), suggesting that the corresponding SGNN pattern reflects differences between the case subsets.

## 4. Discussion

This study presents a proof-of-concept evaluation of a supervised graph-based framework for classifying pre-diagnostic serum proteomic samples from women who subsequently developed PDAC. Rather than treating the graph as a biological interaction network, the SGNN explicitly represents model-derived pairwise statistical information alongside individual protein measurements. To our knowledge, this is the first application of a synolitic graph neural network to pre-diagnostic PDAC proteomic data.

Because longitudinal samples from the same participant may share persistent individual-specific proteomic characteristics unrelated to disease progression, participant overlap between development and holdout cohorts could inflate apparent generalisation if the model partly learns stable participant-specific baselines.

The final 30-protein GATv2 SGNN achieved a mean development-set CV ROC-AUC of 0.753 ± 0.104. After comparable development-only tuning, Random Forest achieved 0.817, XGBoost 0.777, logistic regression 0.772, and SVM 0.732. The present analysis therefore does not demonstrate that the SGNN provides superior within-development discrimination relative to optimised conventional machine-learning approaches. Moreover, because SGNN configuration selection and performance estimation use the same development folds, its CV estimate remains exploratory and potentially affected by model-selection optimism.

In the temporal evaluation, the final SGNN achieved ROC-AUC 0.6435 in the complete 1–2-year holdout and 0.6604 (95% CI 0.5256–0.7888) when restricted to PDAC participants completely absent from development. These analyses provide an exploratory assessment of SGNN performance under temporal separation and participant-independent evaluation. They should be interpreted cautiously because the participant-independent analysis contains only 25 cases and its confidence interval is wide. The present study was not designed to establish comparative superiority of SGNN over conventional models in the temporal holdout.

The minimum-connected sparsification procedure produces genuinely sparse sample-specific graphs while preserving connectivity. In the final 30-node configuration, approximately 73–85 of 435 candidate edges are retained. This sparsification also ensures that topological node descriptors—such as degree, closeness, and betweenness—vary across nodes rather than becoming constant, as they would in a complete unweighted topology. However, the present analysis does not isolate sparsification as the cause of predictive performance, and no such causal interpretation is made. The retained edges remain model-derived statistical quantities rather than physical or biological protein–protein interactions.

It is instructive to contextualise the present results with our two previously published studies on the same UKCTOCS-derived proteomic data. Nené et al. [25] analysed prospectively collected pre-diagnostic UKCTOCS samples using a stacked ensemble classifier and reported AUC 0.91 for samples collected up to one year before diagnosis and 0.85 for samples up to two years before diagnosis. Subsequently, Ney et al. [26] developed a diagnosis-specific ensemble learning model incorporating 44 protein and clinical covariates from both ADEPTS and UKCTOCS symptomatic cohorts, achieving AUC 0.95 in a held-out validation set. Both prior studies differed fundamentally from the present work: ref. [25] pooled both temporal windows and did not use the same temporal stratification, while ref. [26] focused on samples at or close to diagnosis and included clinical covariates (age, gender, diabetes status, BMI, ethnicity) that are excluded here. These design differences preclude direct numerical comparison of performance. In particular, the present study deliberately trains on the 0–1-year cohort and evaluates the 1–2-year cohort separately, and additionally examines a subset of holdout participants entirely absent from development. This represents a more stringent test of temporal and participant-level generalisation than analyses that pool samples across pre-diagnostic windows.

The moderate discrimination observed in the 1–2-year temporal holdout is consistent with the broader challenge of detecting PDAC-related circulating signals at increasing lead times. Prior studies have shown that CA19-9 is significantly elevated up to 2 years prior to diagnosis, but with limited discriminative power in this earlier window [44]. The rapid progression of PDAC from low- to high-tumour stages, with estimated doubling times suggesting progression from stage I to stage IV in approximately one year [12], implies that the proteomic perturbation may be minimal during the 1–2 year pre-diagnostic period, particularly for early-stage disease.

The challenges of early pancreatic cancer detection are well documented [5,6]. Unlike ovarian cancer, where the UKCTOCS trial demonstrated the potential value of longitudinal CA125 monitoring with the Risk of Ovarian Cancer Algorithm (ROCA) [35,45], pancreatic cancer lacks similarly validated screening approaches. The anatomical location of the pancreas, the rapid progression of PDAC, and the heterogeneous nature of the disease all contribute to the difficulty of early detection. Electronic cancer decision support tools (eCDSTs)—such as QCancer, which integrate clinical risk factors and presenting symptoms—offer one avenue for identifying at-risk populations in whom biomarker testing could be applied [46,47]. Our previous work demonstrated that a proteomic biomarker panel significantly outperformed QCancer alone and could serve as a valuable complementary resource for enhanced diagnosis in the clinical setting [26].

The SGNN framework nevertheless provides a different form of model representation from conventional feature-vector approaches. Each sample is expressed as a sparse graph containing individual protein measurements, graph-topological descriptors, and supervised pairwise classifier-derived quantities. This representation permits inspection of highly connected nodes and strongly weighted model-derived edges, although these quantities should be interpreted as properties of the fitted statistical representation rather than as biological interaction measures. The class-average networks in Figure 3 illustrate differences in pairwise classifier-derived graph summaries between PDAC and control samples, but do not establish biological network rewiring or identify the mechanism responsible for classification performance. A dedicated non-graph model receiving the same pairwise information would be required to determine whether graph representation itself provides incremental predictive value. The framework could nevertheless be extended in future to incorporate additional molecular or clinical node and edge information.

GATv2 was selected over GCN within the exploratory development-set configuration search. The dynamic attention mechanism in GATv2 allows the model to adaptively weight the contributions of different protein neighbours, which may be relevant for proteomic data where the predictive relevance of retained graph neighbours varies across samples [41]; however, the present study does not establish that this mechanism confers a predictive advantage. This interpretation is also compatible with our earlier ensemble study, in which the feature sets retained by the diagnosis-specific classifiers differed across models [26], supporting the use of heterogeneous predictive representations.

Several important limitations should be considered. First, the development cohort is small (*n* = 110), producing considerable uncertainty and stochastic variability in cross-validation estimates. The SGNN configuration search and performance estimation used the same outer development folds and no nested cross-validation was undertaken; the development ROC-AUC should therefore be considered exploratory and may be affected by model-selection optimism. Second, although the temporal holdout is temporally separated, it is not fully participant-independent because 25 of the 50 PDAC cases are earlier samples from women represented in development. The participant-independent sensitivity analysis did not show a reduction in the SGNN point estimate (0.6604 versus 0.6435 in the complete holdout), but the confidence interval was wide because only 25 independent PDAC cases were available.

Third, although conventional baselines were subjected to an expanded development-only tuning procedure, the present analysis does not include a non-graph model using exactly the same pairwise SVM-derived information as the SGNN. Consequently, the design does not directly test for a graph-specific predictive advantage, and no such advantage was demonstrated in this dataset. Similarly, in an exploratory comparison, the SGNN showed no incremental discrimination beyond CA19-9 alone, and its value beyond established clinical risk factors was not evaluated. Fourth, UKCTOCS participants in this analysis were exclusively post-menopausal women, so generalisability to men and younger populations requires external assessment. Fifth, clinical covariates—such as diabetes status, BMI and ethnicity—were not incorporated, and comprehensive information on new-onset diabetes was unavailable or incomplete. Sixth, stage at eventual PDAC diagnosis was unavailable, preventing determination of whether the detected signal predominantly reflects biologically advanced but undiagnosed disease or truly early-stage cancer.

Finally, the synolitic edges are supervised model-derived statistical quantities and not biological protein–protein interactions. The current study also does not provide a comprehensive assessment of calibration, precision–recall performance, or sensitivity at clinically relevant high-specificity operating points. These properties should be evaluated in larger, fully independent validation cohorts before clinical utility can be considered.

Future work should prioritise validation in larger, fully participant-independent and more diverse cohorts. Such studies should evaluate calibration, precision–recall performance and sensitivity at clinically relevant high-specificity operating points and should compare incremental performance against CA19-9 and established clinical risk factors. A matched non-graph model using the same pairwise classifier-derived inputs would help determine whether graph representation itself contributes incremental predictive information. Longitudinal sampling could additionally support modelling of within-person trajectories, while graph-explainability approaches could identify model-derived nodes, edges, or subnetworks contributing most strongly to prediction. Any resulting biological hypotheses would require independent validation using pathway, interaction, or experimental data.

## 5. Conclusions

This study provides a proof-of-concept evaluation of synolitic graph neural networks for classification of pre-diagnostic serum proteomic samples from women who subsequently developed PDAC. The final 30-protein GATv2 SGNN achieved a mean development-set 5-fold ROC-AUC of 0.753 ± 0.104. After development-only optimisation, Random Forest achieved a higher mean CV ROC-AUC of 0.817, while XGBoost and logistic regression achieved 0.777 and 0.772, respectively. The present study therefore does not demonstrate superior within-development discrimination for SGNN over conventional machine learning.

In the temporally separated 1–2-year holdout, the SGNN achieved ROC-AUC 0.644 (95% CI 0.526–0.746). Restricting evaluation to the 25 PDAC cases from participants completely absent from development and all 57 controls yielded ROC-AUC 0.66 (95% CI 0.526–0.789). Although the SGNN point estimate was not reduced after excluding overlapping participants, the modest sample size and wide confidence intervals require cautious interpretation.

Overall, SGNN provides a feasible graph-based representation of supervised pairwise proteomic information, but no graph-specific predictive advantage was demonstrated in this dataset, which did not include a head-to-head comparison with a matched non-graph model, and clinical utility is not established. Larger, fully independent external validation, matched non-graph controls, and evaluation against established biomarkers and clinical risk factors are required to determine whether this representation offers incremental value for PDAC early detection.

## Figures and Tables

**Table 1:** Development CV is represented as mean ± SD across the five outer folds. Temporal-holdout performance is calculated once after averaging the five-fold ensemble predictions for each participant; the confidence interval is based on 2000 participant-level stratified bootstrap replicates.

Model	CV Test ROC-AUC	Holdout ROC-AUC
Final SGNN: GATv2, 30 proteins, min-connected sparsification, 10-member ensemble	0.753 ± 0.104	0.644 (95% CI 0.526–0.746)

**Table 2:** Selected conventional machine-learning configurations following development-only tuning. Configurations were selected by mean 5-fold development CV ROC-AUC; feature selection, where applicable, and standardisation were performed within each training fold.

Model	Selected Features	Selected Hyperparameters	Development CV ROC-AUC
Random Forest	97	500 trees, max_depth = 5, max_features = sqrt	0.817
XGBoost	30	depth = 6, lr = 0.05, 300 trees, subsample = 0.8	0.777
Logistic Regression	97	L2, C = 0.01	0.772
RBF-SVM	30	C = 1.0, gamma = 0.01	0.732

**Table 3:** Predictions were obtained by averaging the 10 ensemble-member probabilities within each outer fold and then averaging across the five outer folds for each participant. Confidence intervals are 95% percentile CIs from 2000 participant-level bootstrap replicates stratified by case/control status.

Evaluation Cohort	PDAC Cases	Controls	ROC-AUC	95% Bootstrap CI
Full temporal holdout	50	57	0.6435	0.526–0.746
Participant-independent	25	57	0.6604	0.526–0.789
Participants overlapping development	25	57	0.6267	0.478–0.756

## Data Availability

Access to UKCTOCS samples and associated data is subject to review and approval through the UKCTOCS data-access process. Researchers wishing to undertake secondary analyses are required to submit an application to the UKCTOCS access committee and, where applicable, obtain appropriate ethical approval and enter into the required data-access agreement.
